# The Impact of Fish Predation and Cyanobacteria on Zooplankton Size Structure in 96 Subtropical Lakes

**DOI:** 10.1371/journal.pone.0076378

**Published:** 2013-10-04

**Authors:** Jing Zhang, Ping Xie, Min Tao, Longgen Guo, Jun Chen, Li Li, XueZhen Zhang, Lu Zhang

**Affiliations:** 1 Fisheries College, Huazhong Agricultural University, Wuhan, Hubei, People’s Republic of China; 2 Donghu Experimental Station of Lake Ecosystems, State Key Laboratory of Freshwater Ecology and Biotechnology of China, Institute of Hydrobiology, Chinese Academy of Sciences, Wuhan, Hubei, People’s Republic of China; 3 Nanjing Institute of Geography and Limnology, Chinese Academy of Sciences, Nanjing, Jiangsu, People’s Republic of China; Consiglio Nazionale delle Ricerche (CNR), Italy

## Abstract

Zooplankton are relatively small in size in the subtropical regions. This characteristic has been attributed to intense predation pressure, high nutrient loading and cyanobacterial biomass. To provide further information on the effect of predation and cyanobacteria on zooplankton size structure, we analyzed data from 96 shallow aquaculture lakes along the Yangtze River. Contrary to former studies, both principal components analysis and multiple regression analysis showed that the mean zooplankton size was positively related to fish yield. The studied lakes were grouped into three types, namely, natural fishing lakes with low nutrient loading (Type1), planktivorous fish-dominated lakes (Type 2), and eutrophic lakes with high cyanobacterial biomass (Type 3). A marked difference in zooplankton size structure was found among these groups. The greatest mean zooplankton size was observed in Type 2 lakes, but zooplankton density was the lowest. Zooplankton abundance was highest in Type 3 lakes and increased with increasing cyanobacterial biomass. Zooplankton mean size was negatively correlated with cyanobacterial biomass. No obvious trends were found in Type 1 lakes. These results were reflected by the normalized biomass size spectrum, which showed a unimodal shape with a peak at medium sizes in Type 2 lakes and a peak at small sizes in Type 3 lakes. These results indicated a relative increase in medium-sized and small-sized species in Types 2 and 3 lakes, respectively. Our results suggested that fish predation might have a negative effect on zooplankton abundance but a positive effect on zooplankton size structure. High cyanobacterial biomass most likely caused a decline in the zooplankton size and encouraged the proliferation of small zooplankton. We suggest that both planktivorous fish and cyanobacteria have substantial effects on the shaping of zooplankton community, particularly in the lakes in the eastern plain along the Yangtze River where aquaculture is widespread and nutrient loading is high.

## Introduction

The structure of shallow lake ecosystems could be influenced by human activities such as aquaculture and nutrient enrichment [1]. Freshwater aquaculture is widespread in subtropical and tropical regions, such as China, India, Bangladesh, Vietnam and Thailand [2,3]. Predation by fish is thought to induce shifts in the size structure and species composition of zooplankton [4,5]. In subtropical and tropical shallow lakes, zooplankton communities often comprise small cladocerans, copepods and rotifers [6–8]. These lakes are always found to be dominated by abundant omnivorous-planktivorous fish. Moreover, a low biomass and small mean size of zooplankton are commonly observed in such lakes [9,10]. Experimental studies have documented that a high level of fish predation is responsible for the observed structural patterns of the zooplankton communities in subtropical areas [11,12]. Large-bodied zooplankton species usually consume algae with a wide range of sizes and have a greater impact on phytoplankton than small-sized zooplankton do [13,14]. The absence of large and more efficient filter-feeding zooplankton (e.g., *Daphnia*) results in weak phytoplankton control and low water clarity [10,15].

The aquatic environment could be negatively affected by the waste produced by aquaculture [3]. Many shallow lakes become mesotrophic or eutrophic in subtropical areas because of excessive wastewater discharge and large-scale aquaculture in these lakes [16]. As a result of high nutrient loading, noxious cyanobacterial blooms have occurred in various subtropical shallow lakes [17,18]. It has often been noted that large cladocerans disappear as the proportion of cyanobacteria increases, whereas smaller cladocerans, rotifers and copepods increase [19–21]. Several mechanisms have been suggested to explain this phenomenon, including the low quality of food for zooplankton [22], physical interference with the feeding apparatus [23], and the production of toxins that harm the zooplankton [24]. Large cladocerans are more sensitive to cyanobacteria than the small zooplankton are. This differential sensitivity enables the small-bodied species to dominate during cyanobacterial blooms [20]. The small species can be fed with decomposing cyanobacteria [25]. The biomass of these species was found to be positively correlated with cyanobacterial biomass [26].

Top-down and bottom-up forces are both important in shaping the size structure of zooplankton. As a link between predators and primary producers, the zooplankton size structure reflects the joint influence of nutrient enrichment and aquaculture on freshwater ecosystems. Previous studies have documented that zooplankton size structure could reﬂect the intensity of fish size-selective predation and grazing potential on small phytoplankton [27]. Zooplankton size structure is a sensitive indicator of food web structure [28]. However, a large number of these previous studies were conducted in temperate lakes in Europe and North America, and little is known about zooplankton size structure in subtropical eutrophic lakes in Asian regions. Moreover, negative effects of cyanobacterial species on zooplankton size structure have primarily been found in laboratory studies [24] or small-scale field studies [29]. At the lake ecosystem level, large-scale field surveys in subtropical eutrophic lakes could provide more information on the structure of shallow lake ecosystems.

In the present study, we aimed to assess the effect of predation and cyanobacteria on the zooplankton size structure in shallow lakes along the Yangtze River. The surveyed lakes could be divided into three types, namely, natural fishing lakes with low nutrient loading (Type 1), planktivorous fish-dominated lakes with high nutrient loading (Type 2) and crab culture lakes with a high total phosphorus concentration and high cyanobacterial biomass (Type 3). Additionally, the normalized zooplankton biomass-size spectrum (NBSS) was determined in this study. The NBSS furnishes an approach to the quantification of variation in the zooplankton community structure [30]. The biomass size spectrum has been used to estimate the biological structure and nutrient state of ecological systems and allows for easy comparison across systems [31,32]. Sheldon et al. [33] initially proposed that organisms reached about approximately equal biomass when they were organized in logarithmic size classes, which had been dubbed the “size spectrum” [30]. A consequence of this regularity is that the abundance of organisms decreases with increasing size [30,34]. The flow of energy in pelagic ecosystems is constrained by body size. This phenomenon could be explained by the metabolic theory of ecology (MET), which states that abundance should decrease with size as a function of a one quarter-power allometric relationship with body mass across the entire body size spectrum [35,36]. The slope of the NBSS could be influenced by both the predation pressure and the productivity of lakes [37,38].

Temperature could either directly or indirectly be a major factor that affects the zooplankton size structure [39,40]. To reduce the influence of water temperature on the results, only the data collected during summer have been analyzed in this study. We predicted that lakes with high predation and cyanobacterial biomass will have zooplankton with a smaller size structure and that the abundance of zooplankton will be low in planktivorous fish-dominated lakes and high in crab-dominated lakes. Furthermore, the shape of the NBSS is expected to vary among these three lake types.

## Materials and Methods

### Ethics Statement

No specific permissions were required for the described field studies. The lakes studied are not privately owned or protected in any way and the field studies did not involve endangered or protected species.

### Study Lakes

**Figure 1:**
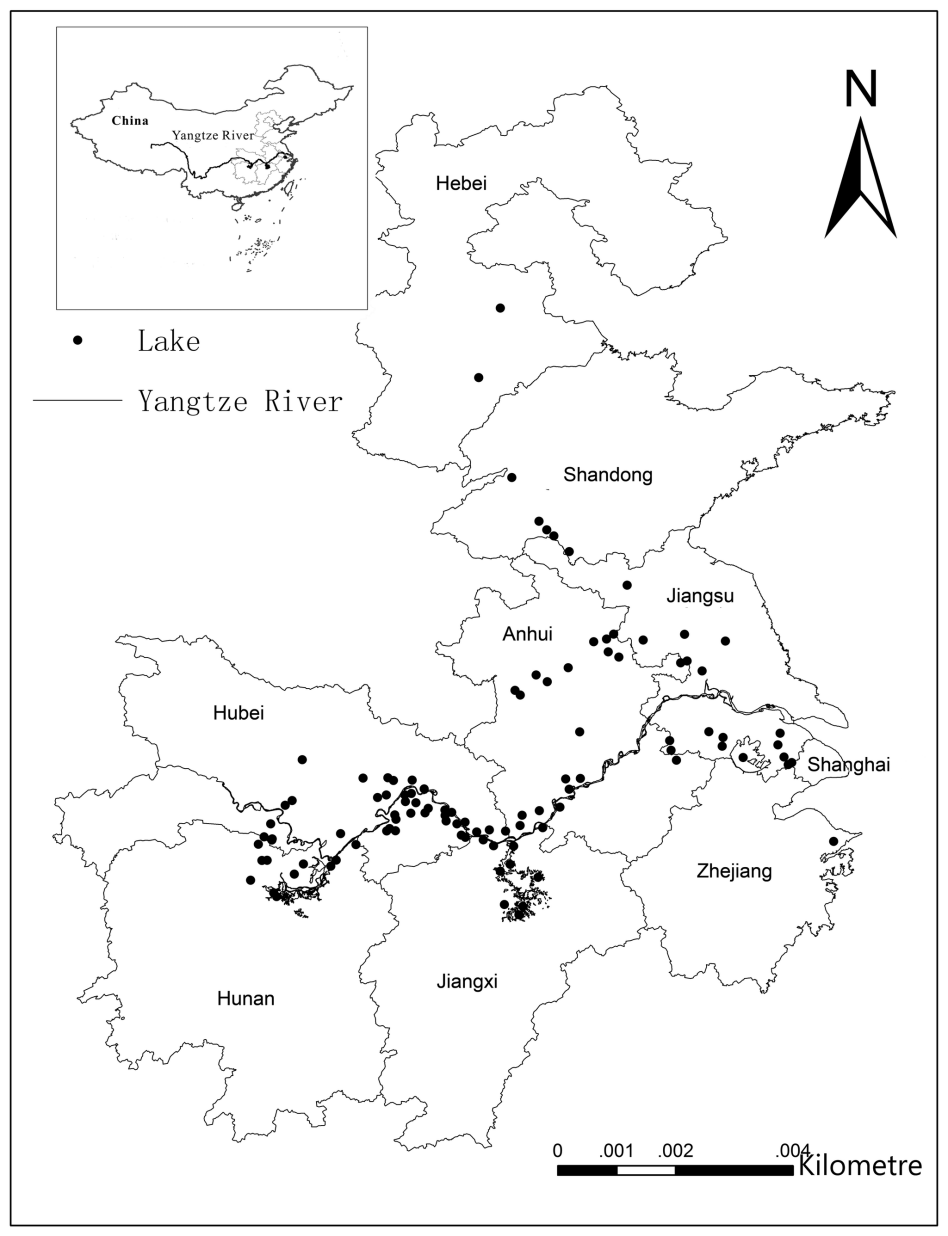
Distribution of the study lakes in the eastern plains area of China.

Zooplankton were initially sampled from a total of 101 subtropical shallow lakes (mean depth, <3 m) located along the middle and lower reaches of the Yangtze River in China (Figure 1). The studied lakes were located at longitudes ranging from 111.7° E to 121.7° E and latitudes ranging from 28.5° N to 38.9° N. A wide range of sizes and nutrient concentrations were observed in the lakes (Table 1).

**Table 1:** The mean and ranges of the abiotic and biotic parameters in three types of lakes and all lakes.

	**Type1 mean**±**STDEV** (**range**)	**Type2 mean**±**STDEV** (**range**)	**Type3 mean**±**STDEV** (**range**)	**All mean**±**STDEV** (**range**)
**TN (mg/L)**	0.76±0.39 (0.30-1.91)	1.32±0.41 (0.39-3.07)	1.73±0.52 (1.29-3.16)	1.18±0.59 (0.30-3.15)
**NO_3_-N(mg/L)**	0.13±0.26 (0.00-1.35)	0.15±0.22 (0.00-0.70)	0.29±0.38 (0.00-1.29)	0.19±0.31 (0.00-1.35)
**NO_2_-N(mg/L)**	0.01±0.01 (0.00-0.03)	0.03±0.04 (0.00-0.13)	0.03±0.05 (0.00-0.17)	0.02±0.04 (0.00-0.17)
**NH_4_-N(mg/L)**	0.24±0.16 (0.05-0.76)	0.36±0.25 (0.06-0.95)	0.27±0.23 (0.09-1.16)	0.28±0.22 (0.05-1.16)
**TP (mg/L)**	0.05±0.02 (0.02-0.12)	0.14±0.09 (0.02-0.43)	0.19±0.10 (0.09-0.49)	0.13±0.09 (0.02-0.49)
**PO_4_-P (mg/L)**	0.01±0.01 (0.00-0.04)	0.02±0.03 (0.00-0.10)	0.03±0.05 (0.00-0.21)	0.02±0.03 (0.00-0.20)
**TN:TP ration**	15±5 (6-28)	11±7 (2-32)	12±7 (4-27)	12±6 (2-27)
**pH**	8.3±0.4 (7.5-9.1)	8.8±0.4 (7.9-9.5)	8.94±0.33 (8.4-9.8)	8.65±0.49 (7.47-9.80)
**DO(mg/L)**	7.27±1.30 (4.24-9.84)	8.70±2.46 (4.25-15.93)	7.32±0.70 (6.23-9.73)	7.92±1.66 (4.24-15.93)
**T(℃)**	29.3±1.8 (24.7-33.1)	29.5±2.0 (25.6-32.8)	28.2±2.0 (28.2-33.5)	28.9±2.0 (24.3-33.5)
**SD(m)**	0.8±0.5 (0.3-2.7)	0.5±0.5 (0.2-2.8)	0.4±0.2 (0.2-0.9)	0.6±0.4 (0.2-2.7)
**Cond(µs/cm)**	252.7±107.5 (61.6-531.3)	385.8±235.7 (74.5-1291.5)	592.9±380.3 (25.2-1524.6)	396.4±291.4 (61.6-1524)
**Cyan(mg/L)**	0.72±1.19 (0.01-6.25)	2.4±2.4 (0.04-7.77)	3.55±4.81 (0.25-26.12)	1.93±3.31 (0.02-26.86)
**TFY(kg/ha)**	357.5±319.4 (23.2-1517)	943.9±450 (418-2450)	490.6±389.3 (63-1644.4)	565.7±450.3 (23.2-2450)
**FY(kg/ha)**	178.7±123.4 (3-450)	744±390.8 (289.2-2047.4)	157.1±150.1 (4.2-622)	334.8±349.5 (3-2047.4)
**Z-BL (mm)**	0.56±0.10 (0.38-0.76)	0.70±0.09 (0.54-0.90)	0.55±0.10 (0.32-0.75)	0.58±0.12 (0.38-0.90)
**Clad-BL(mm)**	0.52 ±0.14 (0.29-0.85)	0.70±0.10 (0.45-0.85)	0.52±0.12 (0.29-0.78)	0.57±0.14 (0.29-0.85)
**Cope-BL(mm)**	0.61±0.10 (0.39-0.81)	0.66±0.11 (0.48-0.90)	0.58±0.07 (0.31-0.74)	0.61±0.10 (0.31-0.90)
**D-BL(mm)**	0.63±0.12 (0.53-0.95)	0.77±0.06 (0.63-0.90)	0.66±0.09 (0.53-0.83)	0.68±0.11 (0.53-0.95)
**ZD(ind/L)**	56.1±39.7 (7.72-151.9)	30.6±17.8 (9.2-87)	136±119.7 (14-561)	75.5±83.5 (7.72-561)

Type1, Type 1 lakes; Type2, Type 2 lakes; Type3, Type 3 lakes; STDEV, standard deviation; TN, Total nitrogen; TP, Total phosphorus; DO, dissolved oxygen; T, Temperature; SD, Secchi depth; Cond, conductivity; Cyan, Cyanobacterial biomass; TFY, Total fish yield; FY, Fish yield; Z-BL, Zooplankto mean body length; Clad-BL, Mean body length of Cladocerans; Cope-BL, Mean body length of Copepods; D-BL, Mean body length of *Diaphonosoma*; ZD, zooplankton density.

### Sampling and Analyzing

Each lake was sampled twice seasonally for zooplankton, water chemistry and phytoplankton during November 2007 and August 2009. The number of sampling stations in these lakes varied from 1 to 35 according to the size of the lake. With a modified 5-L Patalas sampler, we took 20 L of water from the bottom (0-0.5 m over sediment) to the surface (0-0.5 m, surface water) of the lake during the daylight hours. The combined samples were filtered through a 64-µm plankton net and preserved with 5% formalin for further analysis. Crustacean zooplankton was examined at a magnification of 40× with an Olympus microscope (BX41, Olympus, Tokyo, Japan). All individuals in the sample were counted and most organisms were identified to the genus level. Copepods were separated according to life stage into nauplii, copepodites and adults. Species with a body length of >1 mm, 0.5 mm to 1 mm and <0.5 mm were classified as large-, medium- and small-sized cladocerans, respectively (Table 2).

**Table 2:** The list of zooplankton species and their size range found in 96 lakes.

	***Species***	***size range (mm)***
**Large size Cladocerans**	*Leptodora kindtii*	1.45-2.67
	*Side crystalline*	1.07-1.35
	*Simocephalus vetulus*	0.98-1.07
	*Daphnia pulex*	0.90-1.15
	*D. hyaline*	0.92-1.09
**Medium size Cladocerans**	*Diaphanosoma brachyurum*	0.53-0.95
	*Moina micrura*	0.49-0.68
	*Leydigia acanthocercoides*	0.55-0.63
**Small size Cladocerans**	*Ceriodaphnia cornuta*	0.15-0.50
	*Bosmina coregoni*	0.20-0.39
	*Bosminopsis deitersi*	0.22-0.37
	*Ilyocryptus*	0.25-0.50
	*Chydorus ovalis*	0.17-0.48
	*Dunhevedia crassa*	0.35-0.42
	*Alona*	0.19-0.42
	*Craptoleberis testudinaria*	0.28-0.45
	*Camptocercus rectirostris*	0.35-0.51
	*Kurzia latissima*	0.39-0.45
	*Euryalona orientalis*	0.45-0.51
	*Macrothrix hirsuticornis*	0.4-0.53
	*Pleuroxus hamulatus*	0.33-0.48
**Copepods**	*Sinocalanus dorrii*	0.9-1.50
	*Schmackeria forbesi*	0.77-1.23
	*Mongolodiaptomus birulai*	0.97-1.25
	*Neutrodiaptomus alatus*	0.95-1.37
	*Neodiaptomus schmackeri*	0.98-1.27
	*N. yangtsekiangensis*	0.98-1.40
	*Eodiaptomus sinensis*	1.03-1.22
	*Phyllodiaptomus tunguidus*	1-1.5
	*Limnoithona sinensis*	0.39-0.50
	*Eucylops serrulatus*	0.65-0.99
	*Microclops varicaricans*	0.60-0.71
	*Mesocyclops*	0.54-1.17
	*Thermocyclops taihokuensis*	0.59-0.94
	*T. brevifurcatus*	0.60-0.96
	Copepodites	0.22-0.48

Zooplankton was identified according to Sheng [41] and Chiang & Du [42]. The body lengths of cladocerans were measured from top of the head to the base of the tail spine. Copepods were measured from the top of the head to the end of the furca. At least 30 individuals of each species were measured in one sample in each lake. If the number of individuals collected for one species was less than 30, we measured all collected individuals of that species to obtain the mean body length. Biomass dry weight estimates were obtained from the length–dry weight allometric relationships [43,44].

For phytoplankton, a 1-L sample was taken from the same location where the zooplankton sample was collected. The sample was preserved with 1% Lugol immediately after sampling *in situ* and concentrated to 50 mL after sedimentation for 48 h [45]. The identification of phytoplankton species was performed under a microscope at 400 × magnification. A volume of 0.1 ml of the concentrated samples was counted. We used an ultrasonic crusher (JY88-II, Scientiz, Ningbo, Zhejiang, China) to detach cells from cyanobacterial colonies and then counted the single cells. The algal cell dimensions were measured on 10 to 20 individuals for each species, and biomass was estimated by approximation to geometric volumes, assuming that 1µm^3^ is equivalent to 10^−6^ µg fresh weight [46]. Taxonomic identification was conducted according to Hu et al. [47] and John et al. [48].

The water samples were taken from the same location as the zooplankton. The water temperature, Secchi depth (SD), pH, dissolved oxygen (DO) and conductivity of the lakes were obtained *in situ*. The water temperature was measured with a thermometer. The SD was measured with a Secchi disk. The pH, DO and conductivity were measured using a PHB-4PH meter, a JPB-607 DO meter and a DDB-303A meter, respectively. All meters were manufactured by Leici Instrument Co. (Shanghai, China). The total nitrogen (TN), nitrate nitrogen (NO_3_-N), nitrite nitrogen (NO_2_-N), ammonia nitrogen (NH_4_-N), total phosphorus (TP) and phosphate phosphorus (PO_4_-P) were analyzed in the laboratory by the standard methods [45].

Fish yield data were obtained from the Fishery Management Committee of each lake. Domestic Chinese carps (Silver carp, Bighead carp, Grass carp) and crabs were stocked in most surveyed lakes. The fish and crabs would be captured during autumn -winter when the fish were mature and sold in the market. Fish data for each lake were collected during autumn-winter (October-December) when the zooplankton and water samples were taken. Fish were captured throughout the lake by a variety of traditional Chinese methods. Gill nets and dragnets were the principal gear types used to catch fish. The united fish collecting method was used in this study. In this technique, different types of nets were used to catch fish simultaneously. Catching the fish involved three steps. First, a rectangular stow net was placed in an area of deep, open water where fishing could be managed easily. Second, the front of a driving net was laid along the lake bed, and the two sides of the net were pulled by the fishermen. The net was usually wider than the lake width and was used to prevent the fish from escaping from the driving area. The fishermen used different methods, such as swinging bamboo sticks to drive the fish to the stow net. If the lake was too large, it would be divided into several areas for driving the fish. The driving net was made of gill net and was dustpan-shaped. When the fish were driven into the stow net, the fishermen closed it, lifted it above the water, and removed the commercial fish. The mesh size varied according to the type of fish stocked in the lakes. A large mesh size (8-12 cm) was used for the large-sized fish and a small mesh size (3-8 cm) for the small-sized fish. All fish caught by the gear were checked and weighed before they were taken to the market. The total fish yield was the sum of all of the fish caught in the nets. Because Silver (*Hypophthalmichthys molitrix*) and Bighead carp (*Aristichthys nobilis*) were the most dominant planktivorous species, we used the yields of these two species as a proxy to represent the potential predation pressure caused by planktivorous fish.

### Statistical Analysis

**Figure 2:**
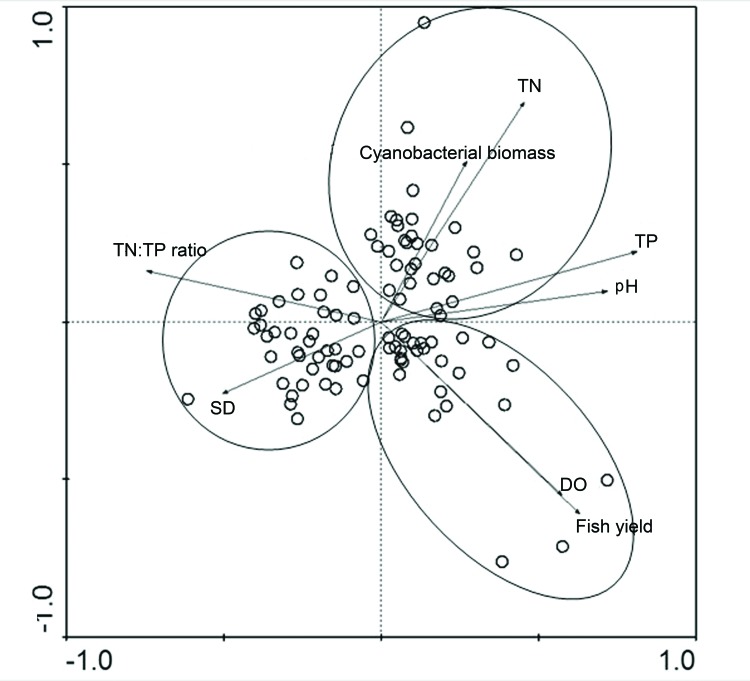
PCA ordination of 8 environmental variables of 96 lakes. All the lakes have been grouped into three clusters.

To understand the relationships among the zooplankton, trophic status, fish and cyanobacteria, only the data collected during the summer were analyzed. The five lakes without summer samples were excluded from the study, and a total of 96 lakes with summer samples were used. A principal component analysis (PCA) was performed to group lakes according to a set of eight environmental variables. These variables were fish yield, DO, pH, total phosphorus (TP), total nitrogen (TN), cyanobacterial biomass, TN/TP ratio, and SD and were used as predictor variables in CANOCO 4.5 (ter Braak and Smilauer 2002). PCA was run on a correlation matrix of centered, standardized and transformed variables. The PCA showed that the first two axes were both signiﬁcant and explained 57.2% of the observed variance in the environmental variables (Figure 2). Based on the results of the PCA analysis we categorized the lakes into three types. The 38 lakes on the left side of the PCA plot were classified as Type 1 and were strongly correlated with SD. The 27 lakes on the right side of the plot were positively correlated with the planktivorous fish yield and were classified as Type 2. The remaining 31 lakes had a strongly relationship with cyanobacterial biomass and were classified as Type 3. The type 1 lakes (38 lakes) functioned as natural fishing lakes and showed high values of transparency. Their TP concentration and the cyanobacterial biomass were the lowest among the three lake types, with mean values of 0.05 ± 0.02 mg/L and 0.72 ± 1.19 mg/L, respectively. The phytoplanktivorous fish yield (primarily *Hypophthalmichthys molitrix* and *Aristichthys nobilis*) ranged from 3 kg·ha^−1^-450 kg·ha^−1^, with a mean value of 178.7 ± 123.4 kg·ha^-1^. Type 2 lakes (27 lakes) showed dense cultures of planktivorous fish (*H. molitrix* and *A. nobilis*, >78% of the total fish yield). The mean planktivorous fish yield was 744 ± 390.8 kg·ha^-1^. Type 3 lakes (31 lakes) were abundantly stocked with crabs (*Eriocheir sinensis*) and had a high nutrient load (TP concentration= 0.19 ±0.10 mg/L) and the highest mean cyanobacterial biomass (3.55 ± 4.81 mg/L) (Table 1, Table 3). Six Type 3 lakes (Taihu Lake, Chaohu Lake, Dianshan Lake, Hongze Lake, Gehu Lake and Gucheng Lake) suffered from cyanobacterial blooms.

**Table 3:** Relative abundance of fish distribution in three types of lakes.

*Type 1*	*Type 2*	*Type 3*	
			RA(%) Mean± STDEV		RA(%) Mean ±STDEV	RA(%) Mean ±STDEV
Cyprinidae						
*Hypophthalmichthys molitrix*			23.2±19.3		33.4±17.1	12.4±6.3
*Aristichthys nobilis*			32.0±22.0		44.8±17.4	17.8±9.3
*Piceous*			0.6±1.4		1.1±1.7	0.7±1.4
*Ctenopharyn odonidellus*			5.8±11.1		3.1±3.8	5.1±7
*Cyprinus carpio*			5.6±9.5		3±3.7	6.3±7
*Parabramis pekinensis*			3.9±4.1		3.3±3.8	1.3±2.2
*Erythroculter ilishaeformis*			1.9±3.1		0.6±1.5	1.1±1.9
Percichthyidae						
*Siniperca chuatsi*			0.9±2.1		1.8±5.3	1.8±3.6
Siluridae						
*Silurus* sp			0.6±2.1		0.2±0.8	1±0.7
Bagridae						
*Pelteobagrus fulvidraco*			1.5±3.3		1.7±3.9	0.5±1.4
Channidae						
*Channa argus*			0.1±0.4		0.2±0.6	0.9±3.4
Grapsidae						
*Eriocheir sinensis*			0.9±19.2		2±4.9	35.6±15.8

RA, Relative abundance; STDEV, standard deviation

Zooplankton sizes were included to identify possible relationships between size and lake characteristics with a PCA. The key environmental variables determined from the PCA were selected for a multiple regression analysis. Multiple regression (a stepwise procedure was used, and variables entered the analysis only if p < 0.05) was performed to identify relationships between zooplankton community parameters (i.e., size) and fish yield, the biomass of cyanobacteria, and the physicochemical environment (TP and SD). We employed separate linear regression analyses to gain further understanding of the single effect of the linkage between zooplankton, fish yield and cyanobacterial biomass. The data used in the statistical analyses were log 10 transformed to meet the assumptions of homoscedasticity and normal distribution of residuals and to increase the coefficient of determination.

The NBSS was generated for each type of lake. We first measured the mean length of every species in each lake and then obtained the biomass dry weight (dry wt) for each species by estimating it from the length-dry weight allometric relationship [43]. Zooplankton individuals were grouped into adjacent size classes in terms of weight on a natural logarithmic (LN) scale. The upper limit of each size class was double the upper limit of the preceding size class. The biomass for each interval was summed, divided by the change in weight occurring between successive size classes [30,49,50], and then plotted against the LN of the upper limit of each size class. The normalized biomass is approximately equal to the density of the organisms in the weight class [30,51]. We used the quadratic regression equation *y* = *a* + 0.5*c* (*x* −*b*)^2^ to fit the data because the size distribution is usually nonlinear [52]. Here, *c* is the curvature of the parabola, whereas *a* and *b* are the *y*- and *x*-coordinates of the vertex, respectively [53]. Parameter *a* and *b* were considered to reflect the total zooplankton biomass and the size of zooplankton at the dome of the parabola, respectively. The relationship between the NBSS parameters and the environmental variables was explored with a linear regression analysis. All of the statistical analyses were performed with SPSS 16.0 for Windows software (SPSS, Inc., Chicago, IL, USA).

## Results

### Zooplankton community structure

**Figure 3:**
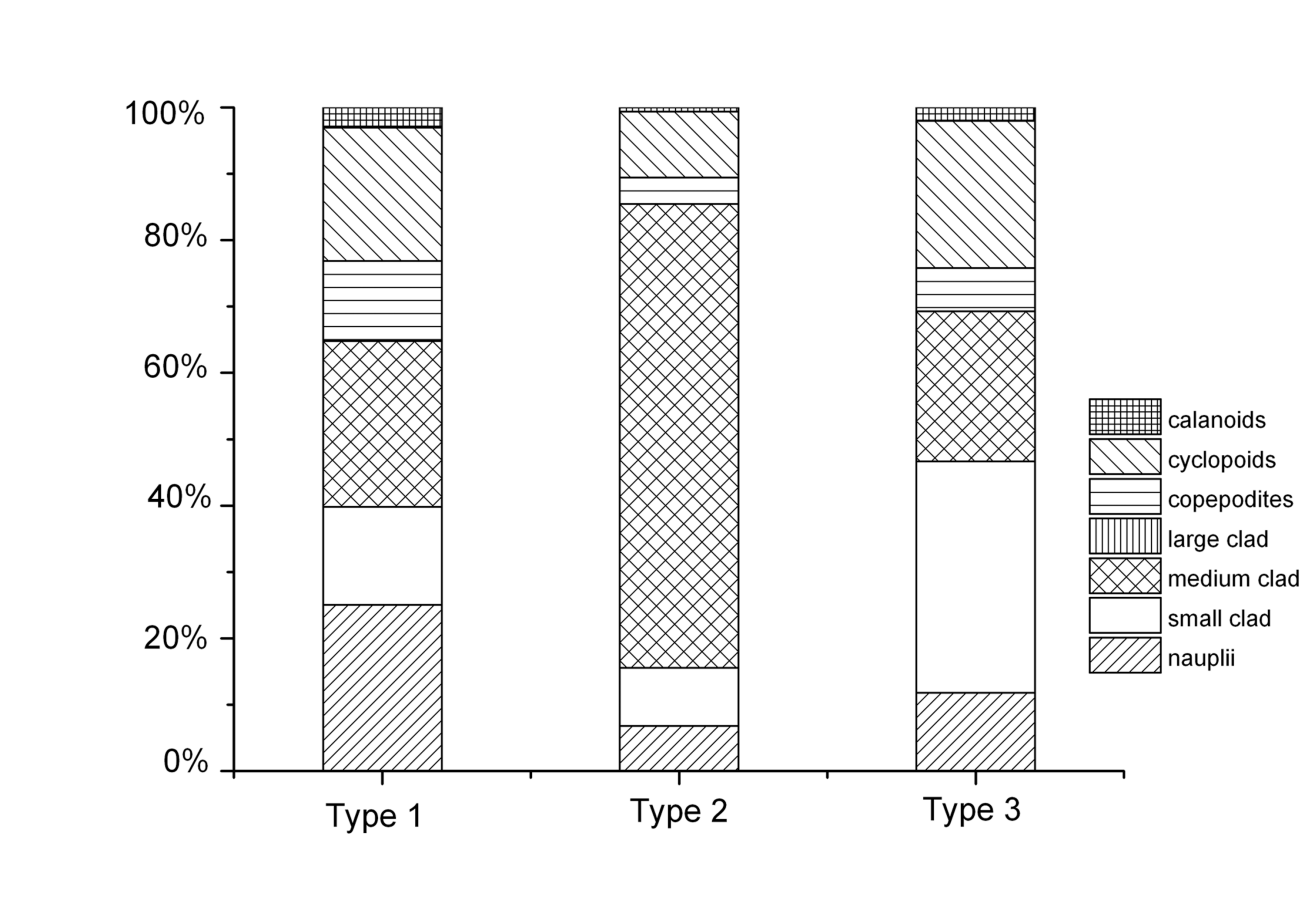
Relative abundances of zooplankton groups in the different types of lakes. Type 1, Type 1 lakes; Type 2, Type 2 lakes ; Type 3, Type 3 lakes.

A total of 35 zooplankton taxa were recorded in all lakes, but the dominant species varied in the three types of lakes. The dominant species in Type 2 lakes were the medium-sized *Moina micrura* and *Diaphanosoma brachyurum*. The small-sized species *Bosmina coregoni* and *Ceriodaphnia cornuta* dominated the Type 3 lakes. These species were more evenly distributed in the Type 1 lakes. *D. brachyurum* was found in almost all of the surveyed lakes (Table 2, Table 4, Figure 3). The zooplankton abundance was the lowest in Type 2 lakes, with a mean value of 30.6 ± 17.8 ind./L, and the highest in Type 3 lakes, with a mean value of up to 136.0 ± 119.7 ind./L (Table 1, Figure 3).

**Table 4:** Mean relative abundance of the dominant species in three types of lakes.

*Dominant species*	*Type 1*	*Type 2*	*Type 3*
	RA(%) Mean±STDEV	RA(%) Mean±STDEV	RA(%) Mean±STDEV
*Ceriodaphnia cornuta*	0.7±0.04	0.1±0.3	5.4±10.2
*Bosmina coregoni*	14.8±19.0	1.1±2.9	21.5±17.5
*Moina micrura*	5.7±13.7	15.7±18.0	9.2±13.0
*Diaphanosoma brachyurum*	15.3±17.6	36.0±19.3	16.3±10.3
*Thermocyclops taihokuensis*	11.6±13.4	17.3±18.7	7.2±6.7
*Mesocyclops*	4.6±5.8	2.9±5.1	4.3±5.0
*Limnoithona sinensis*	3.1±7.3	0.1±0.5	7.7±11.3
*Schmackeria forbesi*	1.4±4.7	0.8±1.4	0.9±0.9
*Sinocalanus dorrii*	0.3±0.8	0.08±0.2	0.4±0.7

RA, Relative abundance; STDEV, standard deviation

The mean body lengths of zooplankton ranged from 0.38 mm to 0.90 mm in the studied lakes, with a mean value of 0.58 ± 0.12 mm. The mean body length of crustacean zooplankton was 0.56 ± 0.10, 0.70 ± 0.09 and 0.55 ± 0.10 mm for Type 1, 2 and 3 lakes, respectively (Table 1). The mean body size of both cladocerans and copepods was higher in Type 2 lakes than in the other two types of lakes. Type 1 and Type 3 lakes showed very similar mean body length for both zooplankton groups (Table 1).

### Results of Statistical Analysis

**Figure 4:**
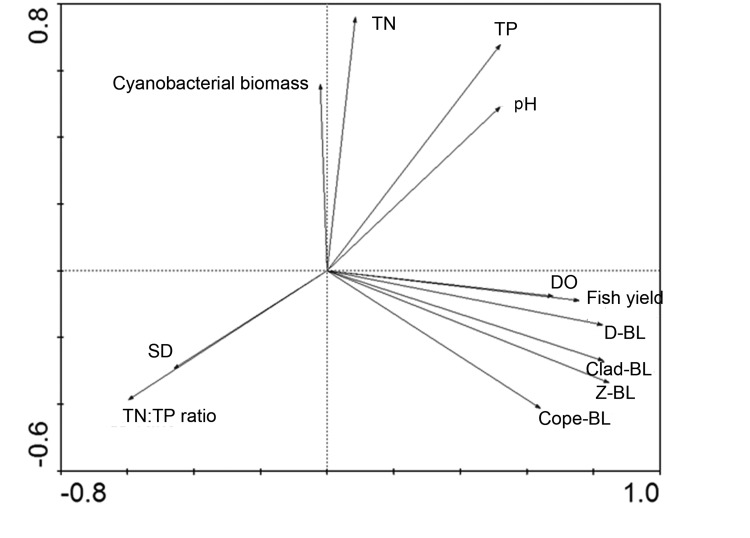
PCA ordination of 8 environmental variables and 4 mean body length variables of 96 lakes. Name of the abbreviation of variables are as denoted in Table 1.

The PCA showed that the first and second axes explained 38.8% and 18.5%, respectively, of the observed variance in the environmental variables (Figure 4). Fish yield was strongly positively correlated with the axis of PCA1 axis, whereas the SD and TN: TP ratio were negatively correlated with this axis. Hence, the PCA1 axis can be interpreted as fish predation pressure, which increased as lake transparency declined. In contrast, cyanobacterial biomass was strongly correlated with PCA2, increasing as the concentration of nutrients increased. The zooplankton mean body length appeared to be strongly positively related to fish yield (Figure 4).

**Figure 5:**
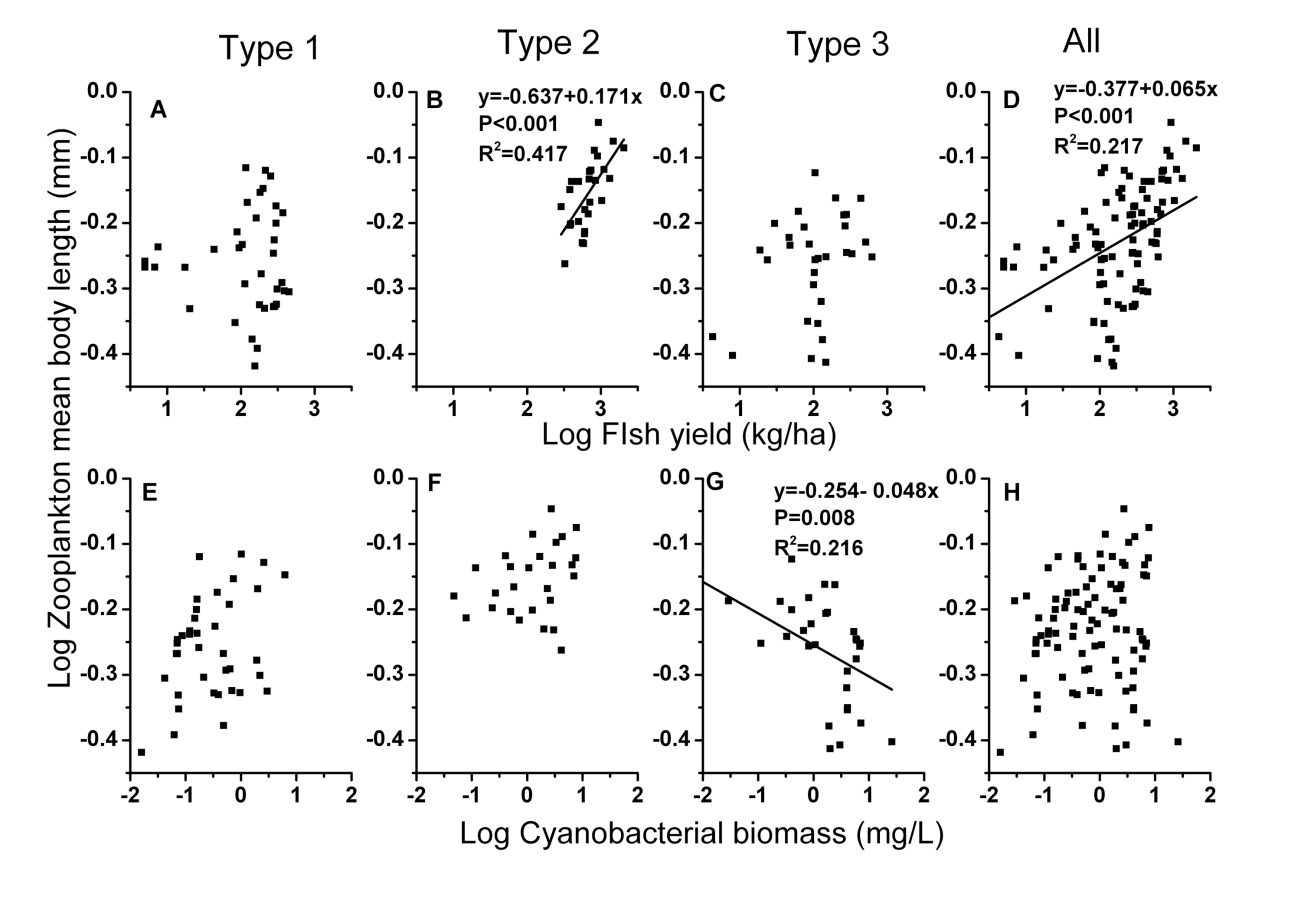
Relationship between zooplankton mean body length and fish yield and cyanobacteria biomass. All, all lakes; Type 1, Type 1 lakes; Type 2, Type 2 lakes ; Type 3, Type 3 lakes.

Based on the results of the PCA ordinations, fish yield, TP, cyanobacterial biomass and SD were selected as initial input parameters for a multiple regression analysis. The multiple regression analysis showed that the fish yield explained the majority of the variation in the mean zooplankton body length. The size of copepods was not as positively correlated with the fish yield as the size of cladocerans (Table 5). The mean body length of the most abundant species, *D. brachyurum*, was positively correlated with the fish yield and cyanobacterial biomass (Table 5). The zooplankton mean size was negatively related to SD, whereas TP had no apparent impact on the zooplankton size if all of the data were analyzed (Table 5). In Type 1 lakes, however, copepods size was negatively related to TP. Stepwise multiple regression performed on Type 2 lakes confirmed that fish yield was the most important predictor of mean zooplankton body size, showing a positive responses for the mean length of zooplankton in a linear regression (R^2^=0.417, p<0.001, Figure 5 B). The decrease in the mean body length of zooplankton with increasing cyanobacterial biomass (R^2^ = 0.216, p = 0.008, Figure 5 G) was caused primarily by the decrease in the cladocerans body length in Type 3 lakes (Table 5).

**Table 5:** The results of stepwise multiple regression analysis.

*Variable*	*Type*	*Coefﬁcients*					*Regression statistics*			
		Intercept	TP	FY	Cyna	SD	F	p	r^2^	n
**Clad-BL**	All	-0.484***		0.086***		-0.121**	18.65	0.008	0.286	96
	1	-0.349***				-0.276**	14.06	0.001	0.281	38
	2	-0.552**		0.14*			5.56	0.026	0.18	27
	3	-0.277***			-0.07**		10.32	0.003	0.263	31
**Cope-BL**	All	-0.3***		0.026*		-0.082**	8.31	<0.0001	0.52	96
	1	-0.478***	-0.172**			-0.141**	8.11	0.007	0.3	38
	2	-0.6**		0.145*	0.04*		7.02	0.004	0.369	27
	3								ns	
**BL,**	All	-0.388***		0.059***		-0.083*	17.11	<0.0001	0.269	96
	1	-0.288***				-0.161**	10.55	0.003	0.227	38
	2	0.637***		0.171***			17.85	<0.0001	0.417	27
	3	-0.254***			-0.05**		7.98	0.008	0.216	31
**D-BL**	All	-0.265***		0.044***	0.032**		16.18	<0.0001	0.258	96
	1	-0.202***			0.046*	-0.112*	6.10	0.005	0.259	38
	2	-0.231**		0.074*	0.026**		7.05	0.004	0.37	27
	3								ns	
**ZD**	All	2.189***		-0.213**	0.214***		12.23	<0.0001	0.22	96
	1								ns	
	2	1.0***	-0.45***				16.96	<0.0001	0.40	27
	3	2.55***		-0.297*	0.189*		10.43	<0.0001	0.43	31

Dependent variables: Clad-BL, Cope-BL, BL, D-BL, ZD. Independent variables included were TP, FY, Cyan. Name of the abbreviation of variables are as denoted in Table 1. All variables were log-transformed. Significance level for coefﬁcients are represented as follows: *p < 0.05, **p <0.01, ***p <0.001. The stepwise multiple regression performed on data when all the lakes taken into consideration(Type All) and divided into three different types of lakes(Type 1 ,2 ,3).

Furthermore, we found that the density of zooplankton was positively related to the cyanobacterial biomass and negatively related to the fish yield in all lakes together as well as in Type 3 lakes (Table 5). In Type 2 lakes, TP was the major factor responsible for the variations in zooplankton abundance, with which it was negatively correlated (Table 5).

### The NBSS of zooplankton

**Figure 6:**
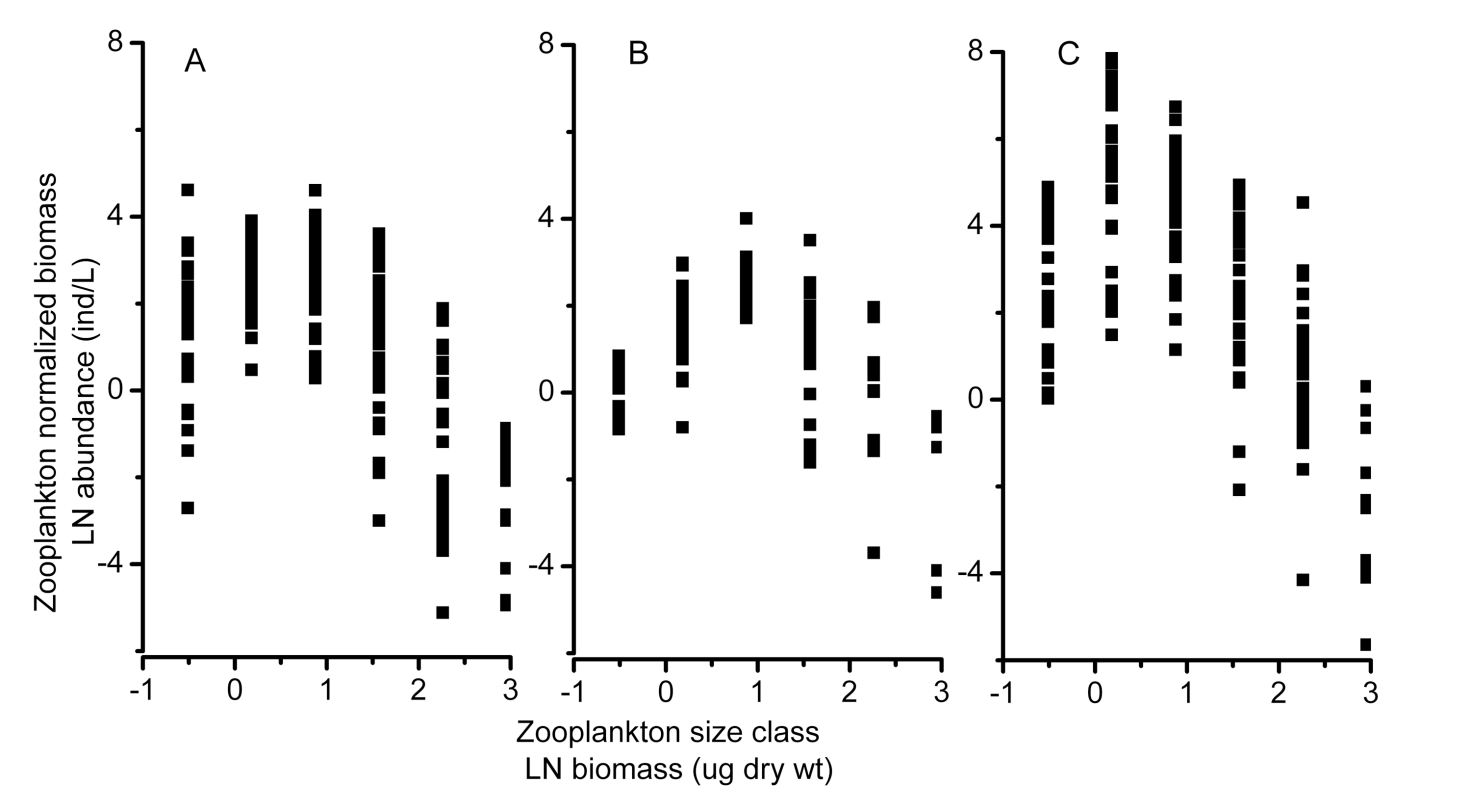
Zooplankton normalized biomass-size spectra (NBSS) for three types of lakes. A, the Type 1 lakes, functioned as natural ﬁshing; B, the Type 2 lakes, which densely stocked with phytoplanktivorous ﬁshes; C, theType 3 lakes, which had the risked to suffer cyanobacterial bloom.

**Figure 7:**
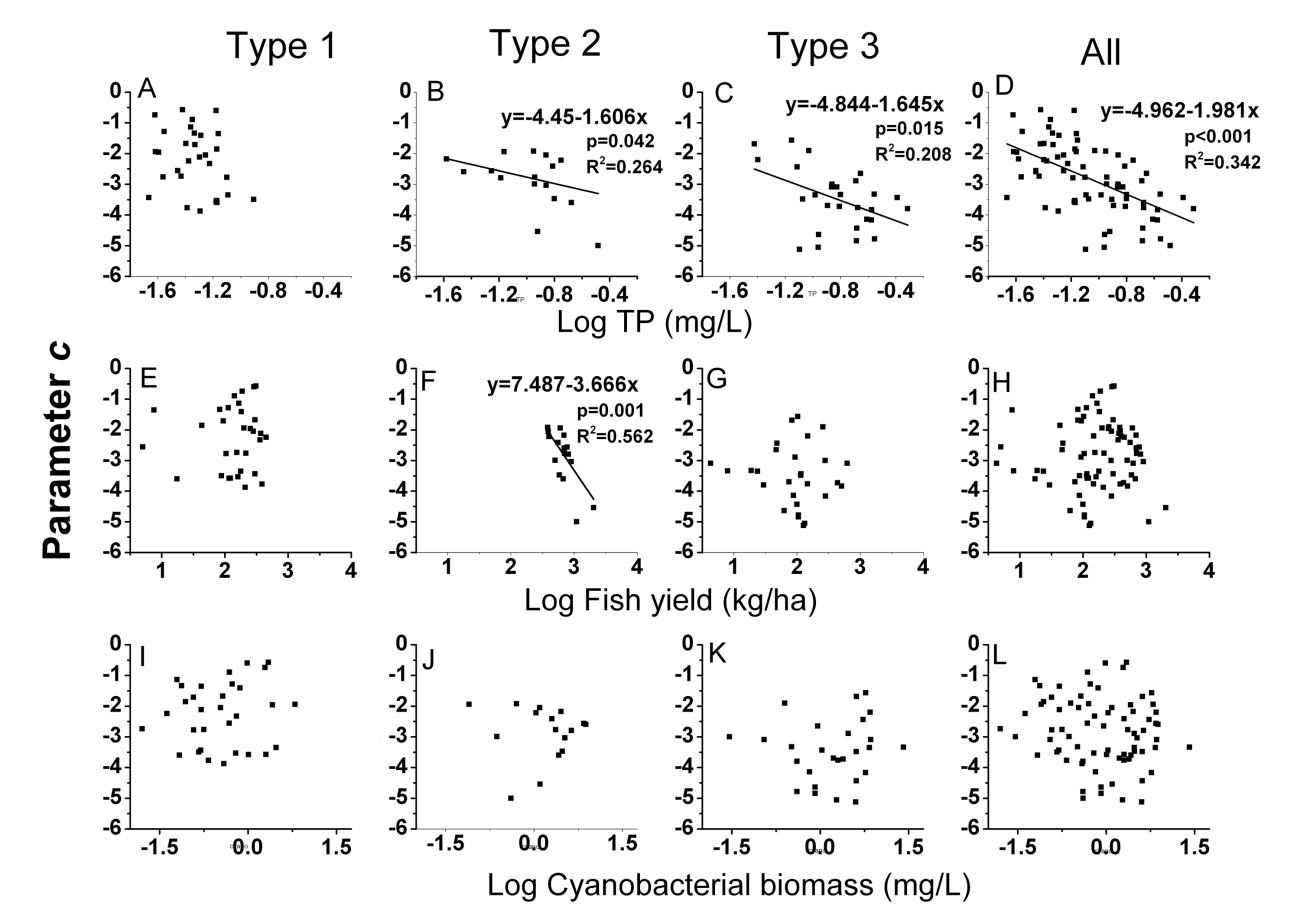
The relation between NBSS parameter *c* and TP, fish yield and cyanobacterial biomass. Name of the abbreviation of types of lakes are as denoted in Figure 5. Lines indicate signiﬁcant regressions (regression p<0.05).

**Figure 8:**
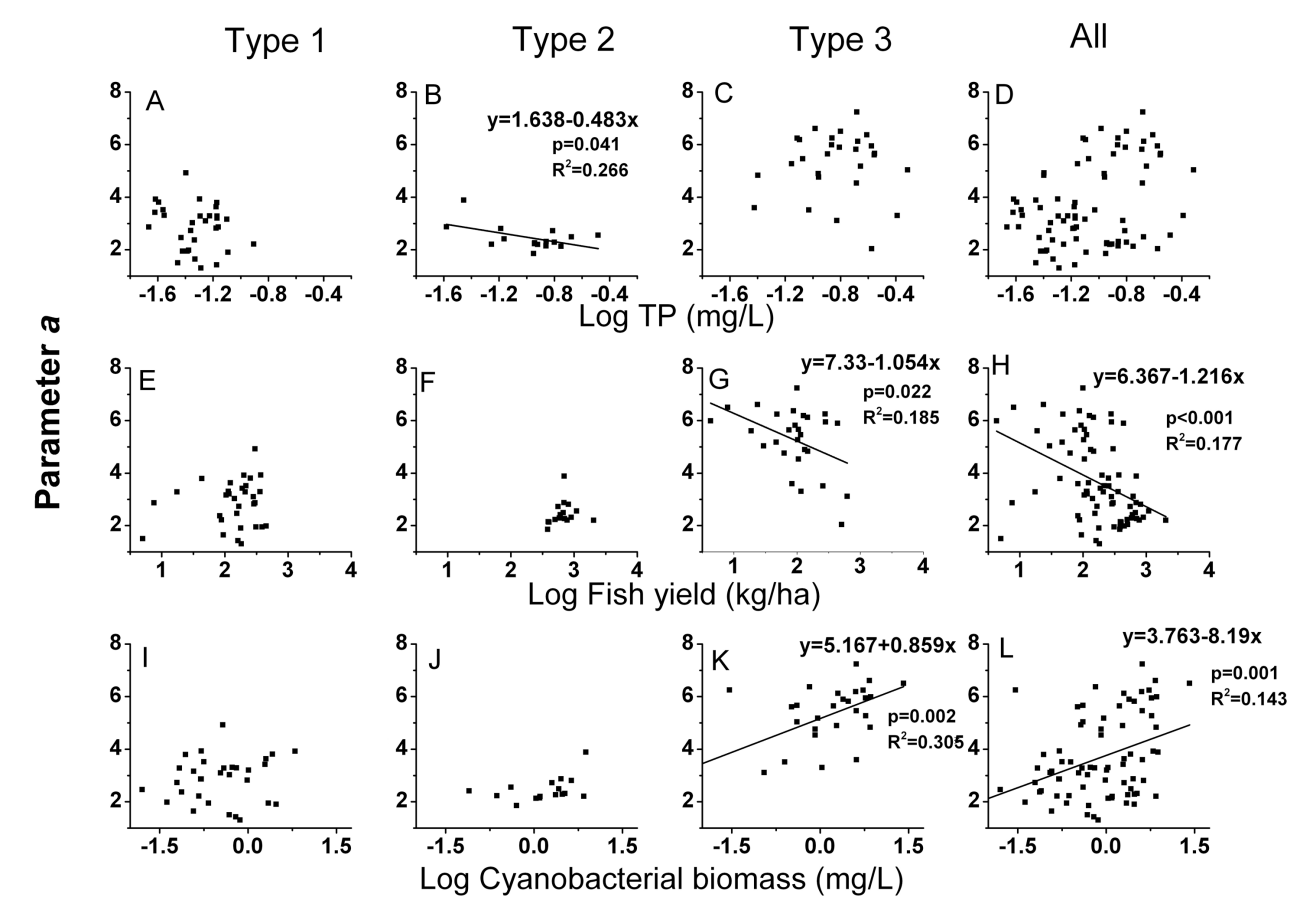
The relation between NBSS parameter *a* and TP, fish yield and cyanobacterial biomass. Name of the abbreviation of types of lakes are as denoted in Figure 5.

The NBSS of zooplankton varied among the three different types of lakes. The normalized biomass of zooplankton showed a unimodal shape against the size class in all three types of lakes, but the curvature of the parabola (parameter *c*) varied among the different types (Table 6; Figure 6). The mean value of parameter *c* was significantly larger in Type 1 lakes. Furthermore, among the 38 lakes of this type included in the study, the size spectra of 7 lakes were better described by a straight line than by a parabola, suggesting that the zooplankton biomass of Type 1 lakes was more evenly distributed across all size classes (Table 6). Different zooplankton sizes in the three types of lakes were reflected in the NBSS, with the Type 2 lakes showing the largest mean value (0.83) of parameter *b* (the *x*-coordinate of the peak). However, 16 out of the 27 Type 2 lakes showed a parabolic fit to the NBSS, whereas the remaining Type 2 lakes had fewer than three size classes available to construct a parabola because only the medium-sized zooplankton were abundant (Table 2, Table 4). Concurrently, the value of parameter *a* (the *y*-coordinate of the vertex) was the smallest in Type 2 lakes (Table 6). This result was a consequence of the lowest zooplankton biomass in these lakes (Table 1). The dome of the NBSS for Type 3 lakes corresponded to small sizes, and the value of parameter *a* for Type 3 lakes was the largest value observed among the lake types (Table 6; Figure 6). The parameter *c* in Type 2 and 3 lakes had a significant negative relationship with TP (Figure 7B, p = 0.042, R^2^ = 0.264; Figure 7C, p = 0.015, R^2^ = 0.208) and fish yield (Type 2 lakes; Figure 7F, p = 0.001, R^2^ = 0.562). The same trend with TP concentration (Figure 7D, p < 0.0001, R^2^ = 0.342) was observed for the parameter *c* of the total data set. Parameter *a* was also negatively related to the TP (Figure 8B, p = 0.041, R^2^ = 0.266) in Type 2 lakes, but was positively correlated with cyanobacterial biomass in Type 3 lakes and in all lakes together (Figure 8K, P =0.002, R^2^= 0.305; Figure 8L, p = 0.001, R^2^ = 0.143).

**Table 6:** The quadratic regression parameters of Normalized zooplankton biomass size spectra for the three types of lakes.

	*Parameter a*		*Parameter b*		*Parameter c*		*R^2^*	*n*
Type	Mean	Range	Mean	Range	Mean	Range	Mean	
1	2.85^a^	(1.31-4.92)	0.26^a^	(-0.72-0.99)	- 2.27^a^	(-3.87--0.57)	0.94	31
2	2.45^a^	(1.85-3.88)	0.83^b^	(0.16-1.17)	- 2.88^b^	(-5.0--1.92)	0.97	16
3	5.27^b^	(2.04-7.23)	0.54^b^	(-0.21-0.96)	- 3.48^b^	(-5.12--1.57)	0.94	27

A parabolic equation is *y*=*a* + 0.5*c*(*x*- *b*)^2^, where c is the curvature of the parabola and *a* and *b* are the *y* and *x*-coordinates of the vertex. Values with different letters indicate significant differences between the three types of lakes (p<0.05).

## Discussion

### Planktivorous fish had no negative effect on zooplankton size structure

Our survey showed that the mean size of zooplankton in the studied lakes was positively correlated with fish yield, suggesting that fish might have a positive effect, rather than a negative effect, on the size structure of smaller crustaceans in subtropical lakes. The mean body length was largest in Type 2 lakes, which were dominated by planktivorous fish. Many previous studies have demonstrated that high levels of fish predation can cause a shift in the predominant zooplankton from large-sized to small-sized [54–56]. Size-selective predation by fish has been a key influence on the size structure of zooplankton communities [5,57]. Therefore, lakes with planktivorous fish were always associated with a smaller mean zooplankton body length and a lower zooplankton biomass than lakes without planktivorous fish [58,59]. However, our results showed that zooplankton in lakes with planktivorous fish had a larger mean body length and that increased fish yield coincided with an increase in mean body size. Furthermore, a unimodal zooplankton size distribution peaking at medium sizes was observed. These results contradict other studies showing that the zooplankton size was negatively affected by fish [54,55]. However, many of the studies focused on lakes that were dominated by large herbivorous cladocerans such as *Daphnia*, which are very vulnerable to fish predation [21]. In contrast, the dominant crustacean in the lakes examined by the current study was *D. brachyurum*, which has been reported to have a greater evasive ability to evade fish predation [60,61]. Likewise, the nutrient concentration and cyanobacterial biomass were much higher in Type 2 lakes than in Type 1 and 3 lakes. Although various studies have shown that planktivorous fish can control cyanobacterial blooms [62,63], the fish also excretes a certain amount of feces into the lake. This input may accelerate the recycling of nutrients and increase the algal biomass [64]. *Cyanobacteria* are poor-quality food for zooplankton [65], but the decomposed cyanobacterial debris and bacteria could be consumed by cladocerans [66]. Thus, we found that the mean zooplankton body length, especially the size of *D. brachyurum*, increased with the biomass of cyanobacteria in Type 2 lakes.

Large-sized cladocerans were rare in our surveyed lakes in summer. This result was consistent with the findings of several previous studies in the (sub) tropics [67,68]. However, large-bodied cladocerans (*Daphnia* spp.) were found and even maintained at relatively high densities in tropical areas, especially in artiﬁcial reservoirs without effective planktivorous fish [69,70]. The sediment in several of the lakes that we surveyed has been used successfully to hatch *Daphnia* spp. [71,72]. Therefore, a lack of large-bodied cladocerans might suggest that strong fish predation eliminates these animals from the community, with negligible or positive effects on the remaining size classes. In doing so, planktivorous fish may release medium-sized species from competition and cause them to become the absolute dominant species in aquaculture lakes. We also found a negative relationship between zooplankton density and fish yield. We assumed that under high predation pressure combined with the effect of cyanobacteria, although the abundance of the zooplankton was decreased by the fish, such predation had a positive effect, rather than a negative effect, on the size structure of the zooplankton.

### High Cyanobacterial Biomass Caused a Decline in the Size Structure of the Zooplankton Community

The expected decrease in zooplankton body size with increasing cyanobacterical biomass was not evident if all of the lakes were considered. However, Type 3 lakes, which had the highest average cyanobacterial biomass and showed instances of noxious blooms in several lakes, showed stronger negative relationships between zooplankton mean body length and cyanobacterical biomass. Zooplankton body size was smallest in Type 3 lakes. We attributed these results to the high abundance of the small zooplankton associated with high cyanobacterial biomass. These findings suggested that the zooplankton communities tended to shift toward smaller species with increasing cyanobacterial biomass. These findings were consistent with previous studies showing that small zooplankton groups dominated as a result of high densities of cyanobacteria in lakes [73,74]. Cyanobacteria may affect zooplankton in several ways, including nutritional insufficiency [22], clogging of the feeding apparatus [75] or toxicity[24]. . Small cladocerans may show a survival advantage and appear to be less affected by cyanobacteria, as they may avoid consumption of large colonies of cyanobacteria and are more tolerant to the cyanobacterial toxin [72,76]. However, the length of copepods appears to have been less affected by cyanobacteria. Copepods can behave flexibly toward significant populations of cyanobacteria, either by size-selective feeding on colonies or by distinguishing between toxic and nontoxic particles [77]. Ghadouani et al. [76] have stated that zooplankton might not be negatively affected during the first stage of moderate dominance by cyanobacteria but could suffer a serious negative influenced if cyanobacteria reached high biomass levels. This result was confirmed in the lakes with planktivorous fish, where a positive relationship was found between body length and cyanobacterial biomass. Fish might be the primary factor determining the size structure of zooplankton in these lakes, as we previously argued. We suggested that cyanobacteria could cause a decline in zooplankton size if they reached a high biomass level and predation by fish was low.

### Fish and cyanobacteria were responsible for the greater curvature in the NBSS of zooplankton

The NBSS of zooplankton is a useful tool for estimating the effects of fish and cyanobacteria on zooplankton size structure. The NBSS varied among the three different types of lakes. According to the MET, the abundance of organisms should decrease with size as *M* ^−3/4^ within a trophic level, where *M* is the body mass [36]. On a logarithmic scale, therefore, the abundance of organisms should show a negative linear relationship with body mass. However, the shape of the NBSS of zooplankton is always better described by a parabolic function [52,53]. More curvature (more negative values of parameter *c*) in the size spectrum was observed in Type 2 and Type 3 lakes, with the peak of the unimodal shape at medium and small sizes. This finding indicated that relative increases in medium- and small-sized species occurred in Type 2 and Type 3 lakes, respectively. This result is consistent with the findings of previous studies, which showed that parameter *c* was more negative in productive lakes [78]. Additionally, TP was significantly negatively related to the values of parameter *c* in our studied lakes. This result is consistent with the findings of Finlay et al. [37]. The factors behind the parabolic curves and the lake productivity were more difficult to explain than expected. We assumed that high fish predation and cyanobacterial biomass would be responsible for the variation in the NBSS of zooplankton. We observed that parameter *c* decreased with increases in fish yield in Type 2 lakes, and the biomass-size spectra distribution peaks at medium size. Parameter *a*, which was considered to reflect the total zooplankton biomass, showed a positive relationship with the cyanobacterial biomass in Type 3 lakes. Field research has shown that small cladocerans were enhanced by cyanobacteria bloom [26]. Therefore, as cyanobacterial biomass increased, a relative increase in small zooplankton was responsible for the greater curvature in the zooplankton NBSS in Type 3 lakes. Parameter *a* was significantly lower in Type 2 lakes and was negatively related to the TP. This observation was in line with the results found for the relationship between zooplankton abundance and the nutrient concentration (Table 5), indicating that zooplankton biomass did not increase with increasing nutrient loading. This result is in contrast to the findings of other studies [79]. High predation may be the major factor responsible for the lower value of parameter *a*. The results of the zooplankton NBSS analysis supported the predictions that high cyanobacterial biomass had a negative effect on zooplankton size structure and that the high level of predation kept the abundance of the zooplankton at a low level.

### Insights for lake management and aquaculture

Previous studies have shown that intensive fish farming has significant impacts on the aquatic environment. Aquaculture wastes can cause the deterioration of water bodies due to nutrient enrichment [80]. What are the long-term consequences for aquatic ecosystems of the degradation of water resulting from aquaculture? Our results for zooplankton mean body length and the NBSS analyses might indicate that zooplankton community structure is already depauperate as a consequence of a long-term history of impacts of eutrophication and aquaculture. Donghu Lake, one of our survey lakes, has been long-term monitoring. It has been an aquaculture lake since the 1960s. The annual fish yield markedly increased in the 1980s. The lake has been stocked heavily with planktivorous fish (Silver carp and Bighead carp) and became eutrophicated as a result of increased sewage disposal [81-84]. Drastic changes have been found in the zooplankton community. The density of the large-bodied cladocerans *Daphnia* was observed to decrease from 28.3 ind./L in 1971–1986 to 0.9 ind./L in 1987–1996, and the dominant cladocerans shifted from *Daphnia* to *Monia* and *Diaphonosoma* (Table 7). The intensive culture of planktivorous fish would cause a dramatic change in the zooplankton community. The disappearance of large-sized cladocerans would result in a weak impact on the phytoplankton and would cause low water clarity. This finding implies a need to control the extent of aquaculture and the composition of the cultured fish as well as a need to reduce nutrient loading.

**Table 7:** The history changes in annual crustacean zooplankton community of Lake Donghu.

*Years*	*Fish yield (kg/ha)*	*TP (mg/L)*	*Cladocera Mean density (ind/L)*	*Copepoda Mean density (ind/L)*	*Daphnia Mean density (ind/L)*	*Dominated species*	*Sources*
1962-1970	95.41	0.08^a^	27	29	–	*Daphnia hyalina* Diaphanosoma	[81–83]
1971-1986	450.13	0.428^b^	35.76	78.59	28.3	*Daphnia hyalina* Diaphanosoma *Mesocyclops leuckarti*	[82,83]
1987-1996	945.13	0.144^c^	10.49	52.06	0.9	*Diaphanosoma Moina Cyclops vicinus*	[82–84]
2008	400	0.177	20.3	0.3	0	*Moina*	This study (summer data)

— missing data; a, the data during 1956-1957; b, the data during 1980-1985; c, the data 1995-1997, we used these data as a proxy to present the nutrient concentration during the three phases of the lake Donghu.
